# Supplementary material for: Applications of Boolean modeling to study the dynamics of a complex disease and therapeutics responses
Source: Front Bioinform. 2023 Jun 1;3:1189723. doi: 10.3389/fbinf.2023.1189723 (PMC10267406; doi:10.3389/fbinf.2023.1189723)
Supplement: Supplementary file 1 [file DataSheet1.pdf]

Supplementary Material

1 SUPPLEMENTARY TABLES AND FIGURES

2 SELECTED DIAGRAMS FROM PD MAP

We include the following pathway diagrams from PD map:

- Dopamine transcription pathway (fig. S1)
- PPARGC1A (fig. S2)
- FOXO3 activity pathway (fig. S3)
- PI3KAKT signalling pathway (fig. S4)
- mTOR pathway (fig. S5)
- PRKN signalling (fig. S6)
- TCA cycle (fig. S7)

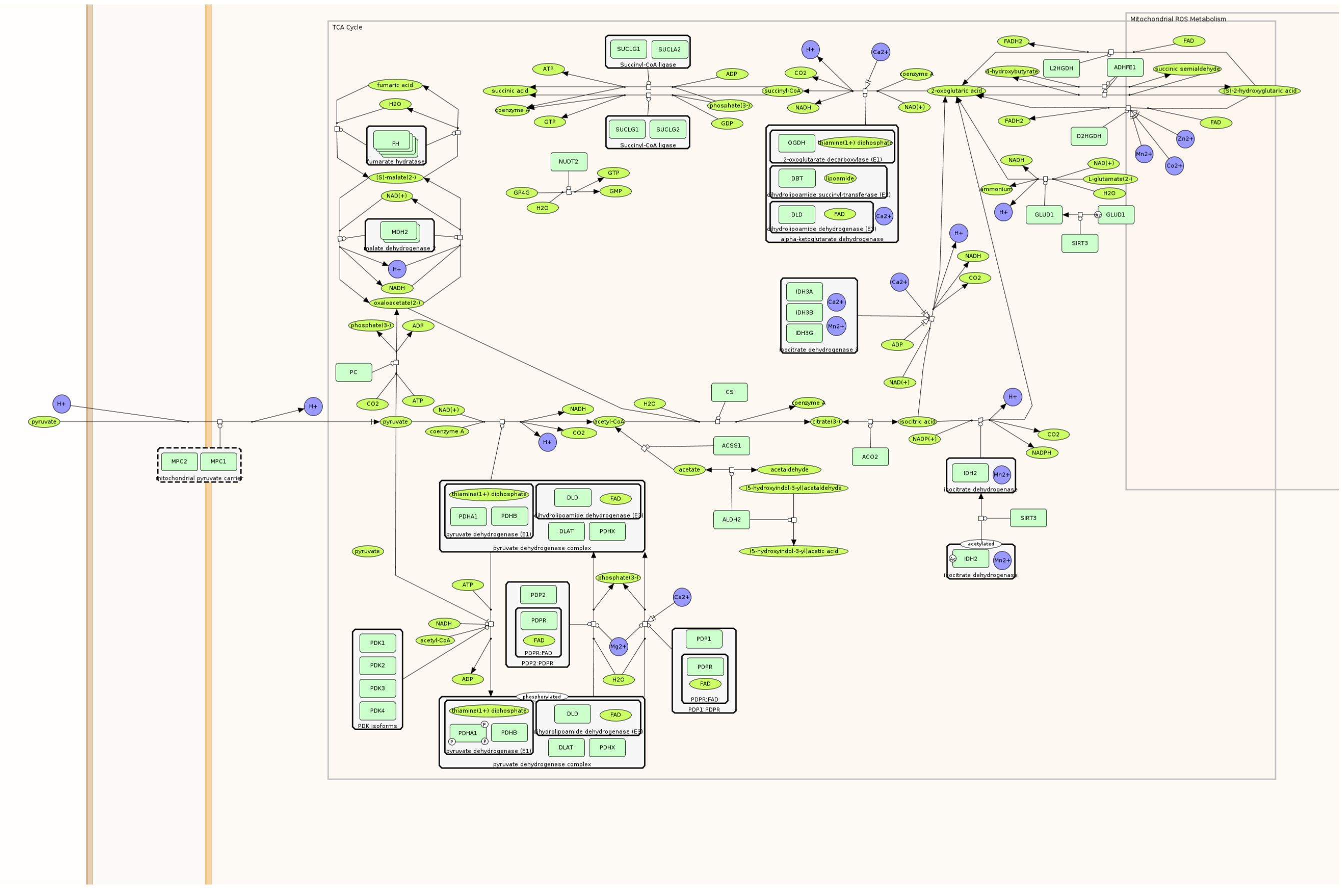

Figure S1: Dopamine transcription pathway

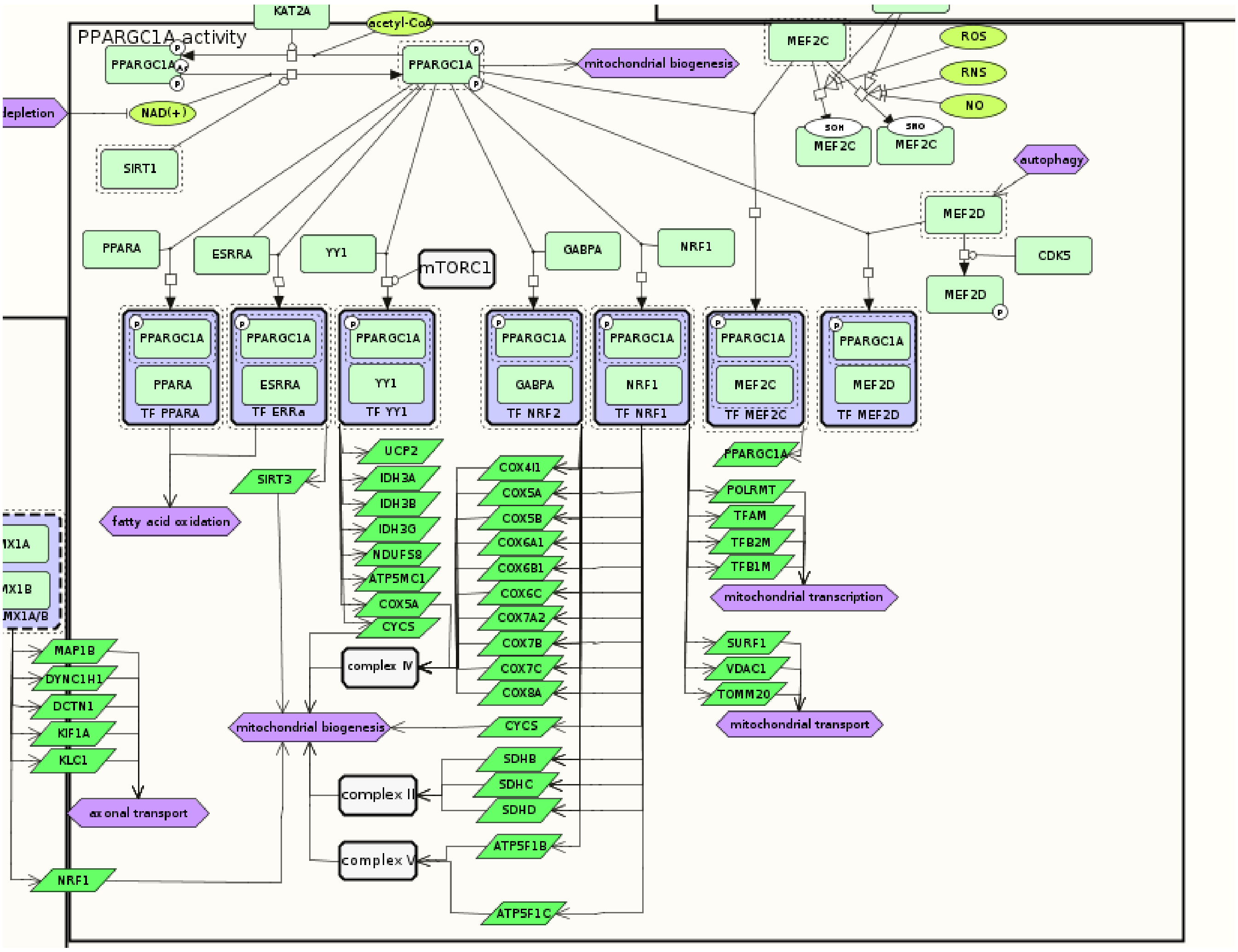

Figure S2: PPARGCA1

3 THE SIMULATION GRAPHS FROM BOOLEAN SIMULATIONS

This section details the simulation graphs that resulted from Boolean models and Probabilistic BMs simulation. All simulation graphs are also available in the gitlab repository .

The examples from simulation graphs from CellCollective platform are shown in figs. S8 to S11. Interactive demo and guidance on how to construct and simulate the models is available at `https://cellcollective.org/`.

4 THE SENSITIVITY AND STRUCTURAL ANALYSIS

Distances computed between the original and perturbed attractors are summarized in table S1 for knockouts and table S2 for overexpressions.

Identifying high betweenness centrality molecules with low sensitivity is important as it suggests the presence of compensatory paths. This information can be used to develop targeted interventions to disrupt the function of the pathway in pathological conditions. The results of our study highlight potential intervention points in different pathways, such as Wnt, ppargc1a, mTOR, Foxo3, and dopamine.

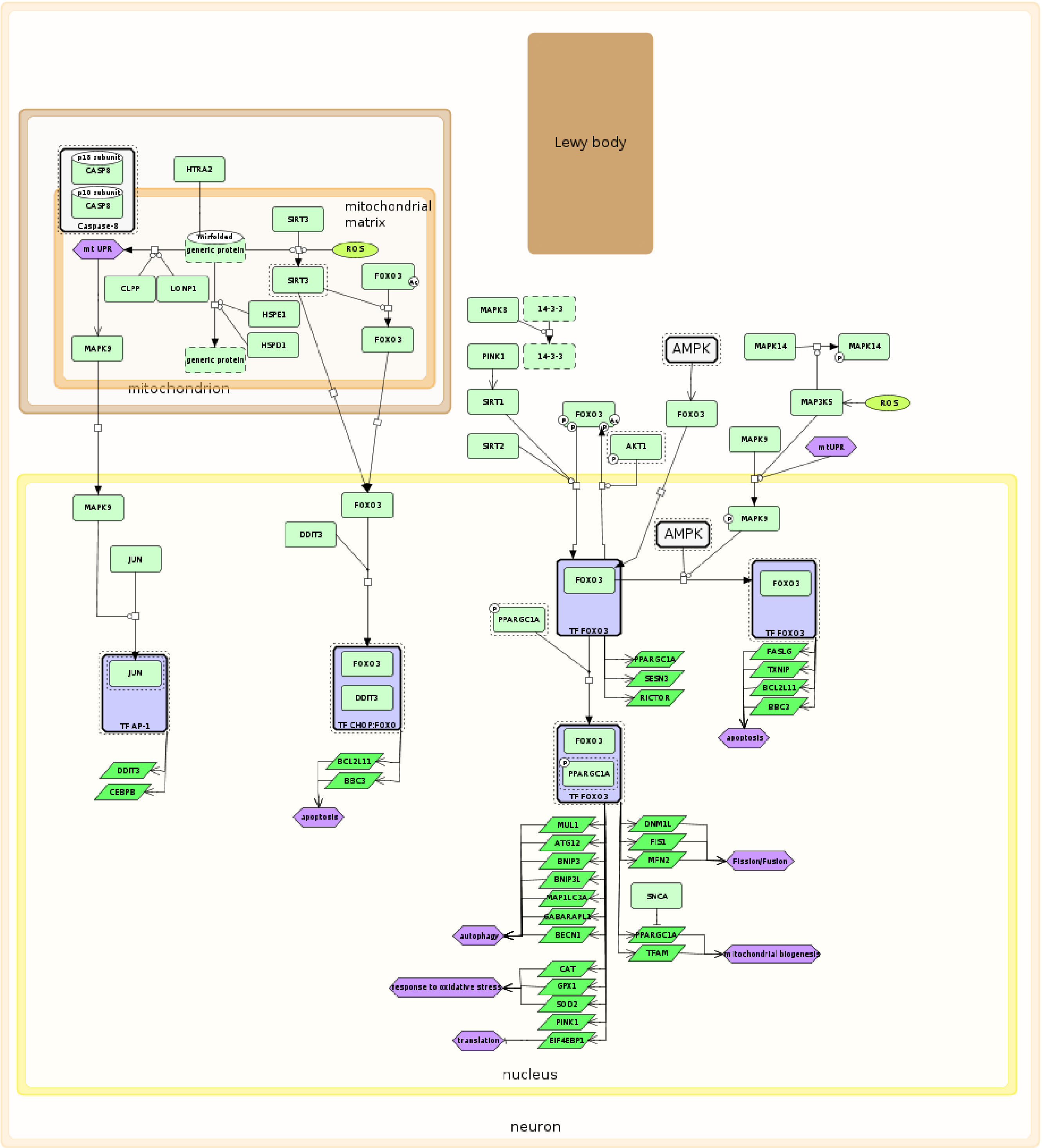

Figure S3: FOXO3 Activity pathway

For instance, in the Wnt Pi3kAKT pathway, molecules such as GRM3, TGFB1 rna, IGF1, and INS compartment can compensate for the absence of PDPK1, RPS6KB1 phosphorylated, and IRS1 phosphorylated. In the PPARGC1 pathway, different subunits of the complex itself, various mitochondrial genes, and transcription factors such as TF NRF1 complex can compensate for the loss of Complex IV. In the mTOR pathway, compensatory molecules include other upstream signals, SIRT1 and PPARGC1 for

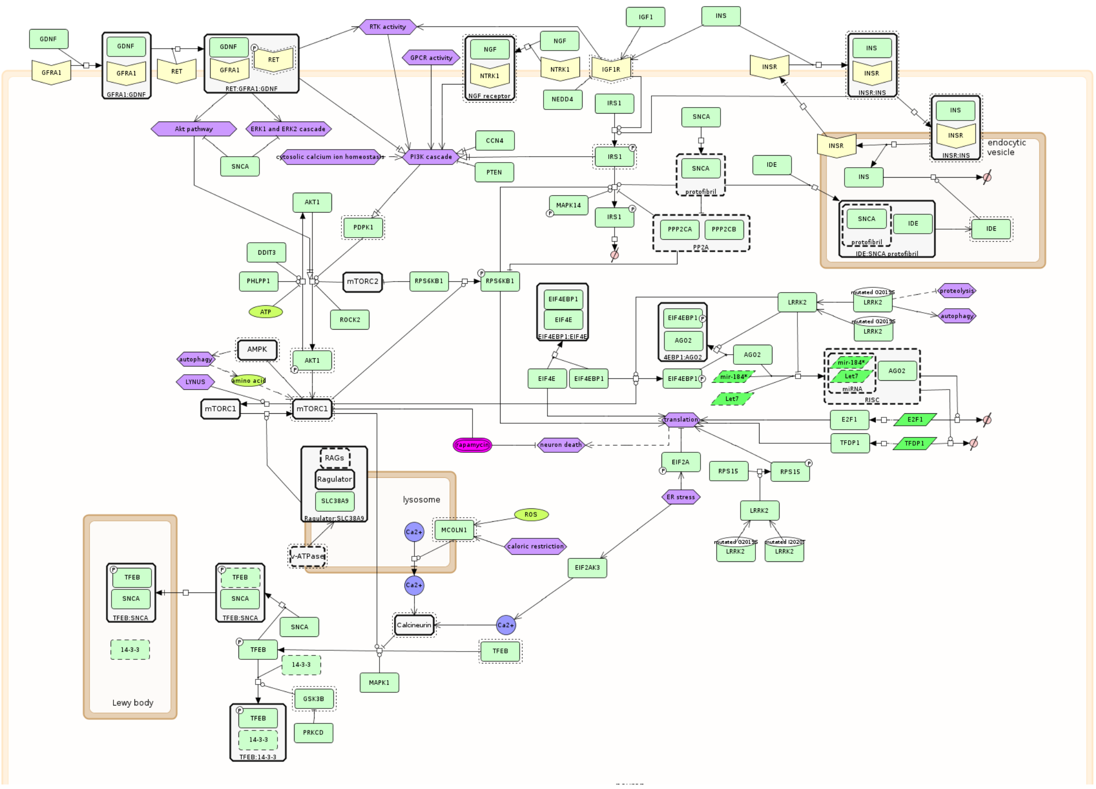

Figure S4: PI3KAKT signalling pathway

AMPK complex neuron, other upstream signals for TSC1 TSC2 complex neuron, and other molecules involved in autophagy and lysosome function for SEN2. Similarly, in the FOXO3 pathway, TF CHOP FOXO complex and other pro-apoptotic molecules such as BCL2L11 rna and FASLG rna can compensate for the absence of BCL2L11 rna and BBC3 rna, and other forms of FOXO3 or other transcription factors for FOXO3 acetylated phosphorylated and MAPK9 phosphorylated. Finally, compensatory molecules for the dopamine transcription pathway involve other molecules in dopamine metabolism, neuron survival, and retinoic acid synthesis phenotypes such as SLC18A2 rna, GDNF rna, and TF PITX3 complex for BDNF rna, SLC18A2 rna, SLC6A3 rna, DRD2 rna, ALDH1A1 rna, and TF PITX3 complex for TH rna, and retinoic acid and TF NR4A2 complex for RXRA.

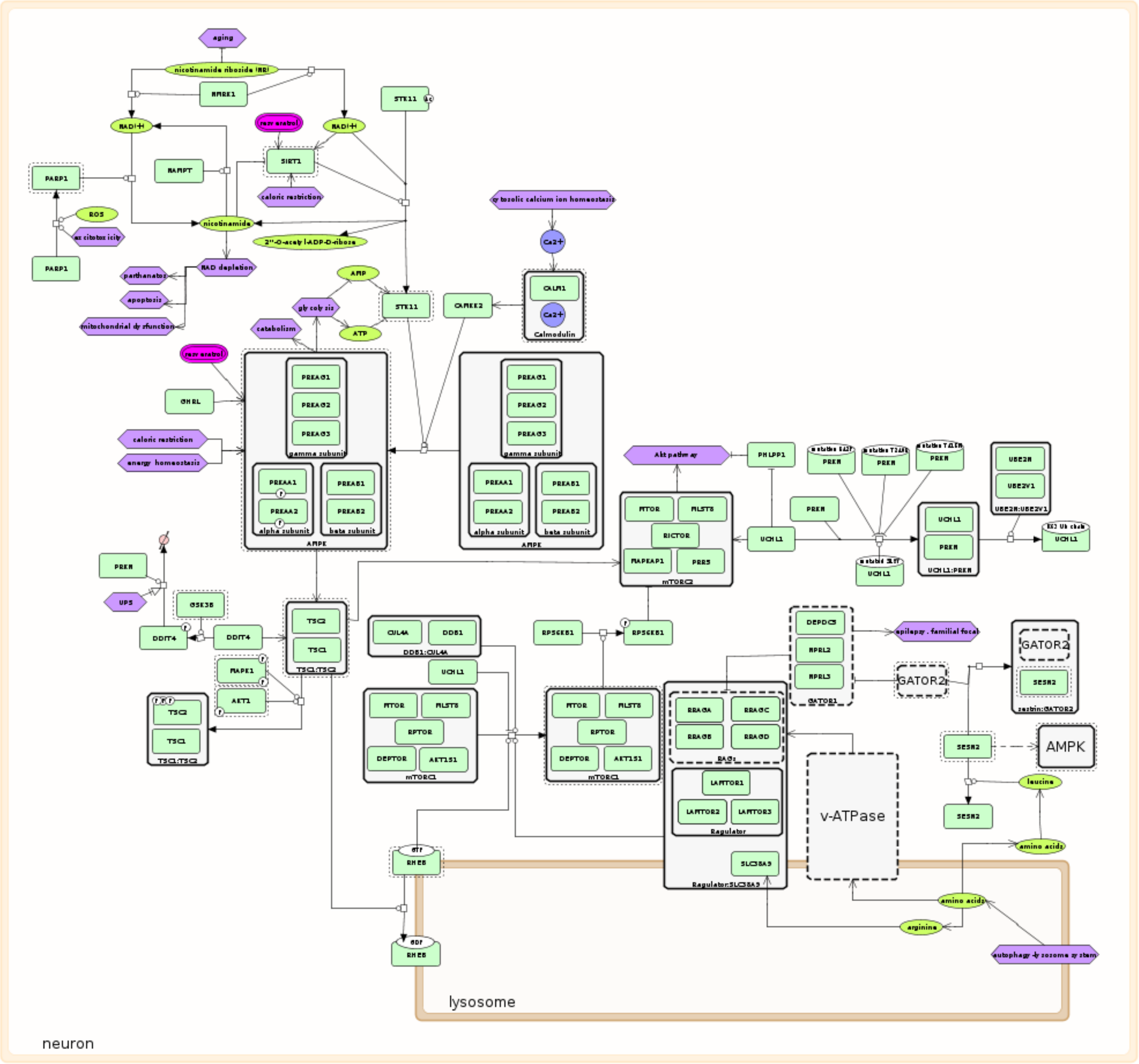

Figure S5: mTOR pathway

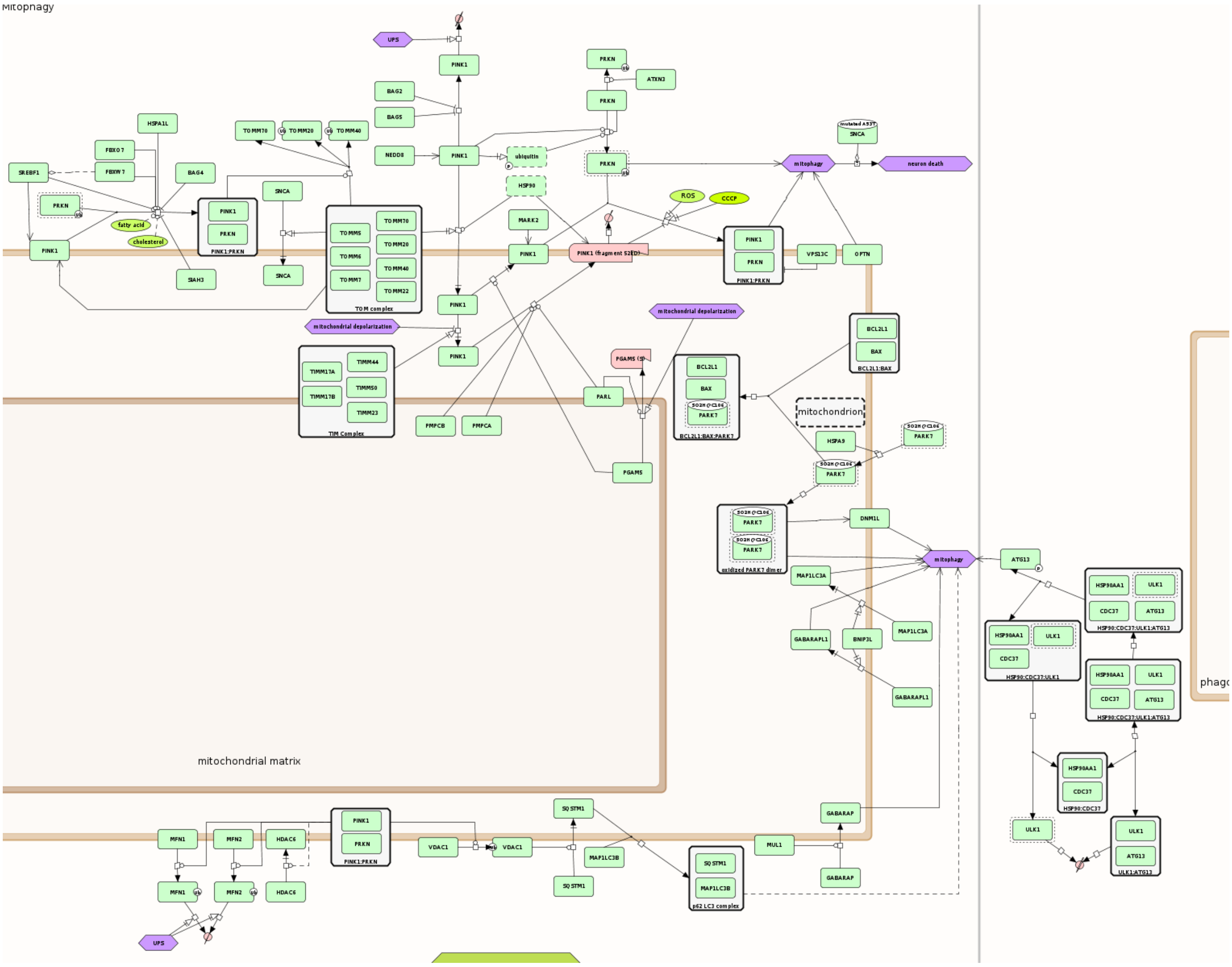

Figure S6: PRKN signalling pathway

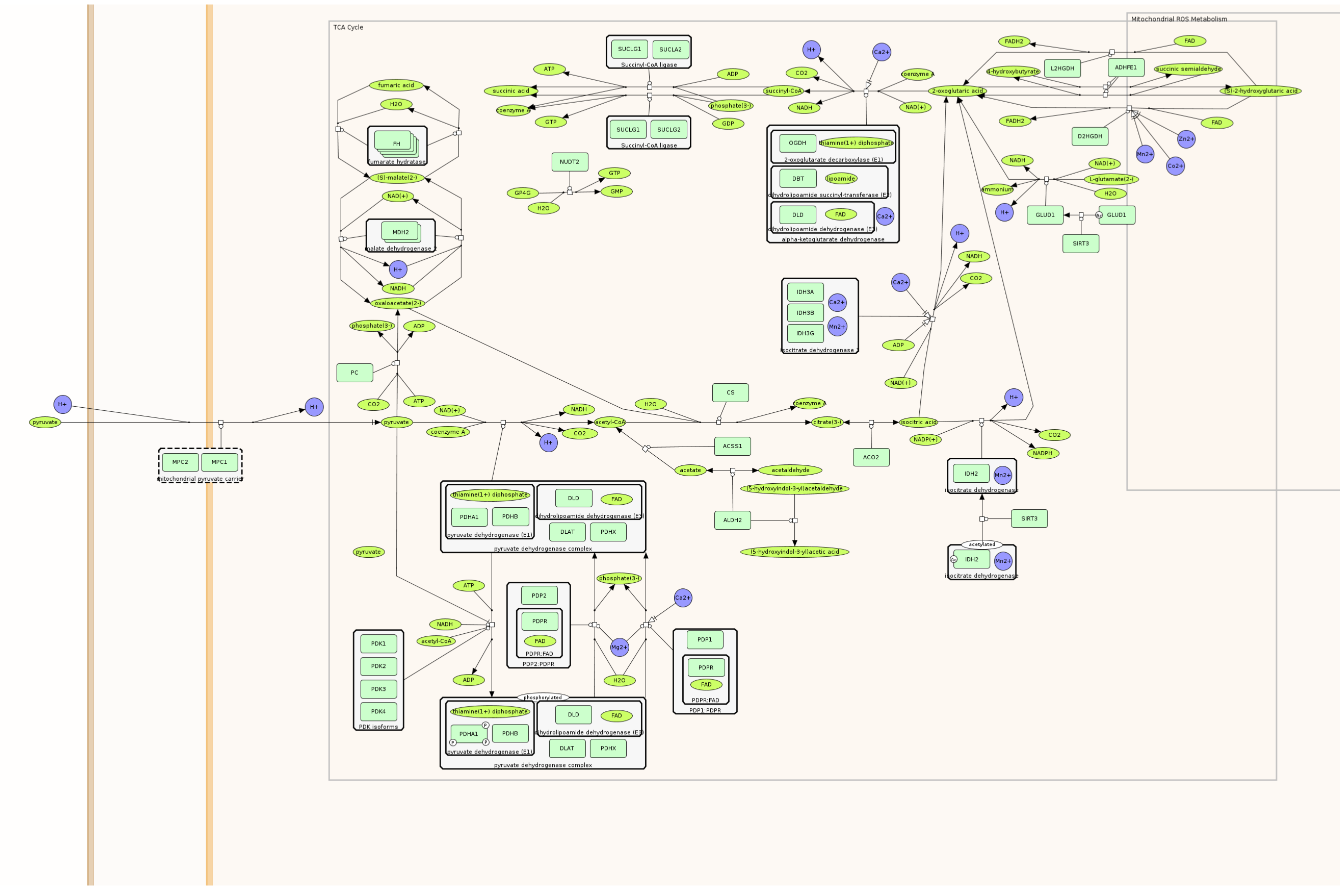

Figure S7: TCA cycle

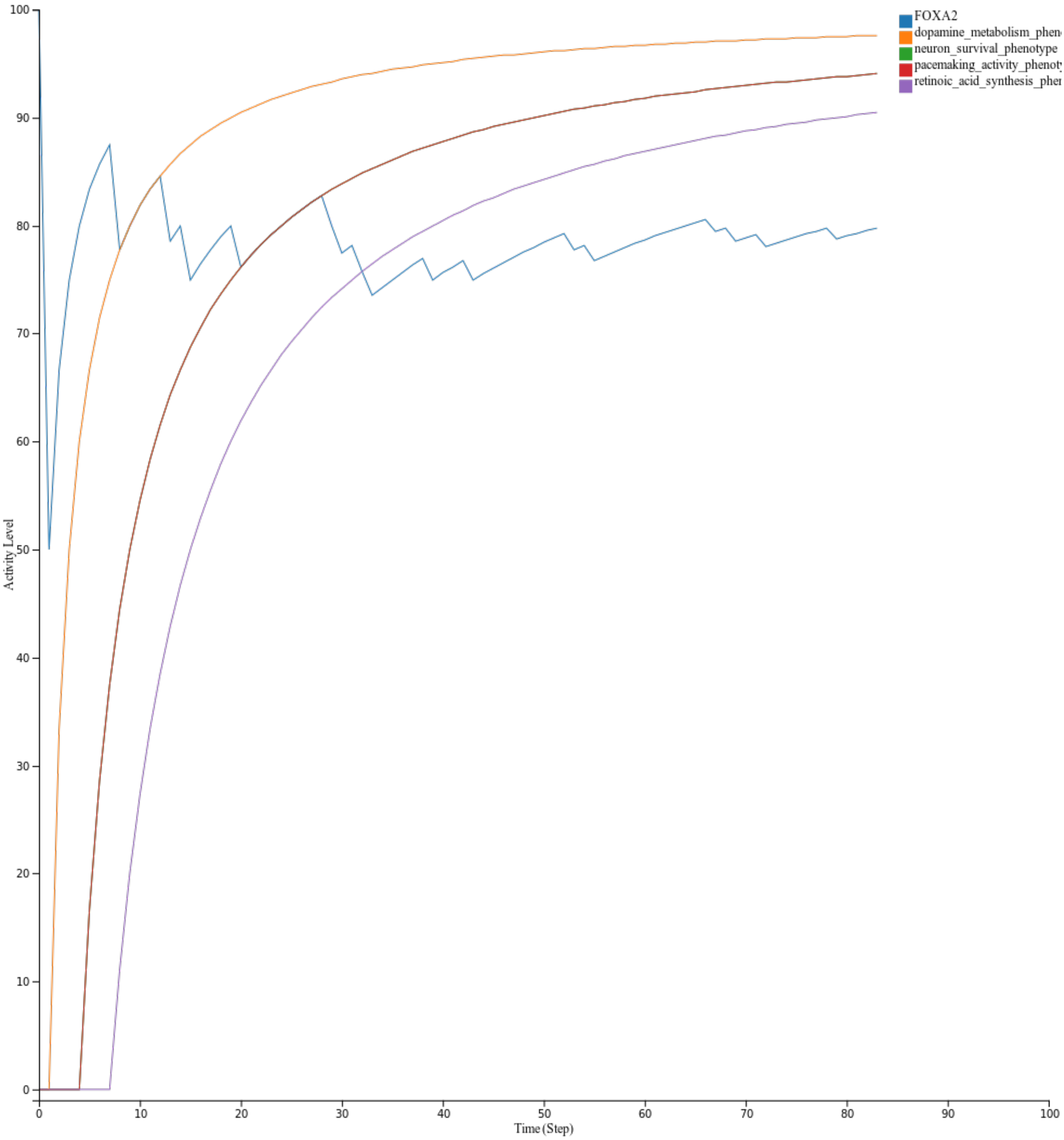

Figure S8: Dopamine transcription simulation

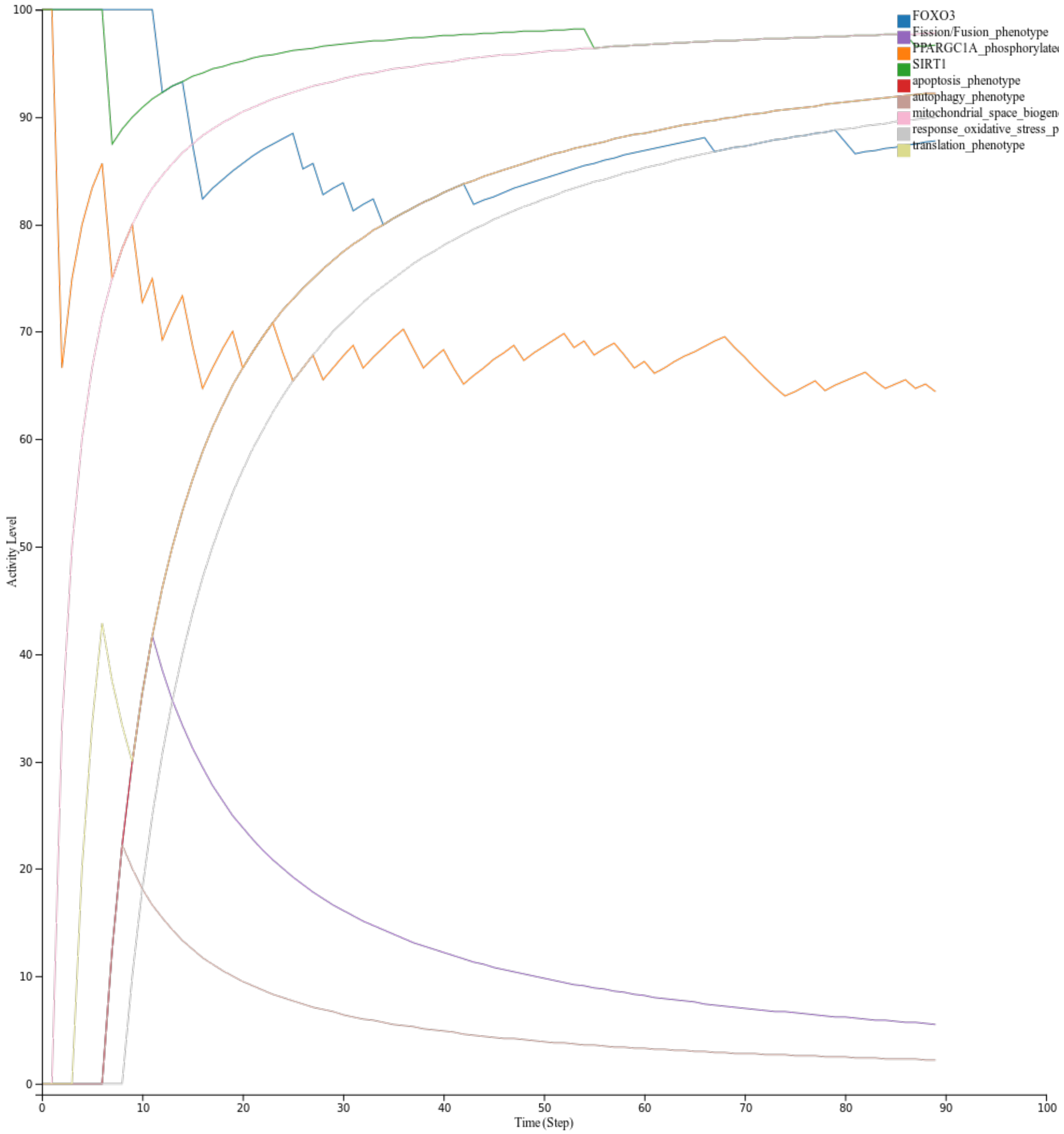

Figure S9: FOXO3 simulation

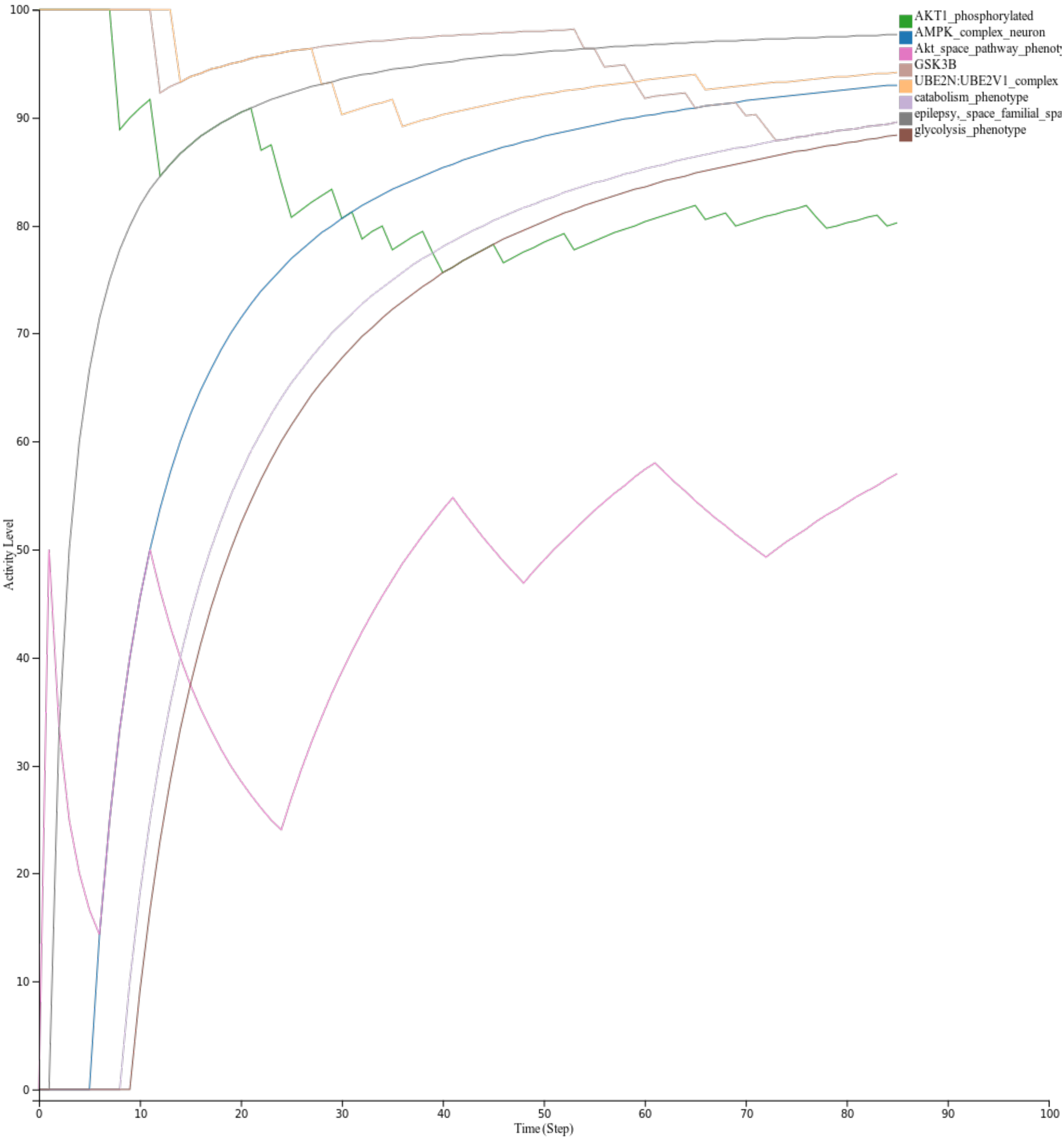

Figure S10: mTOR simulation

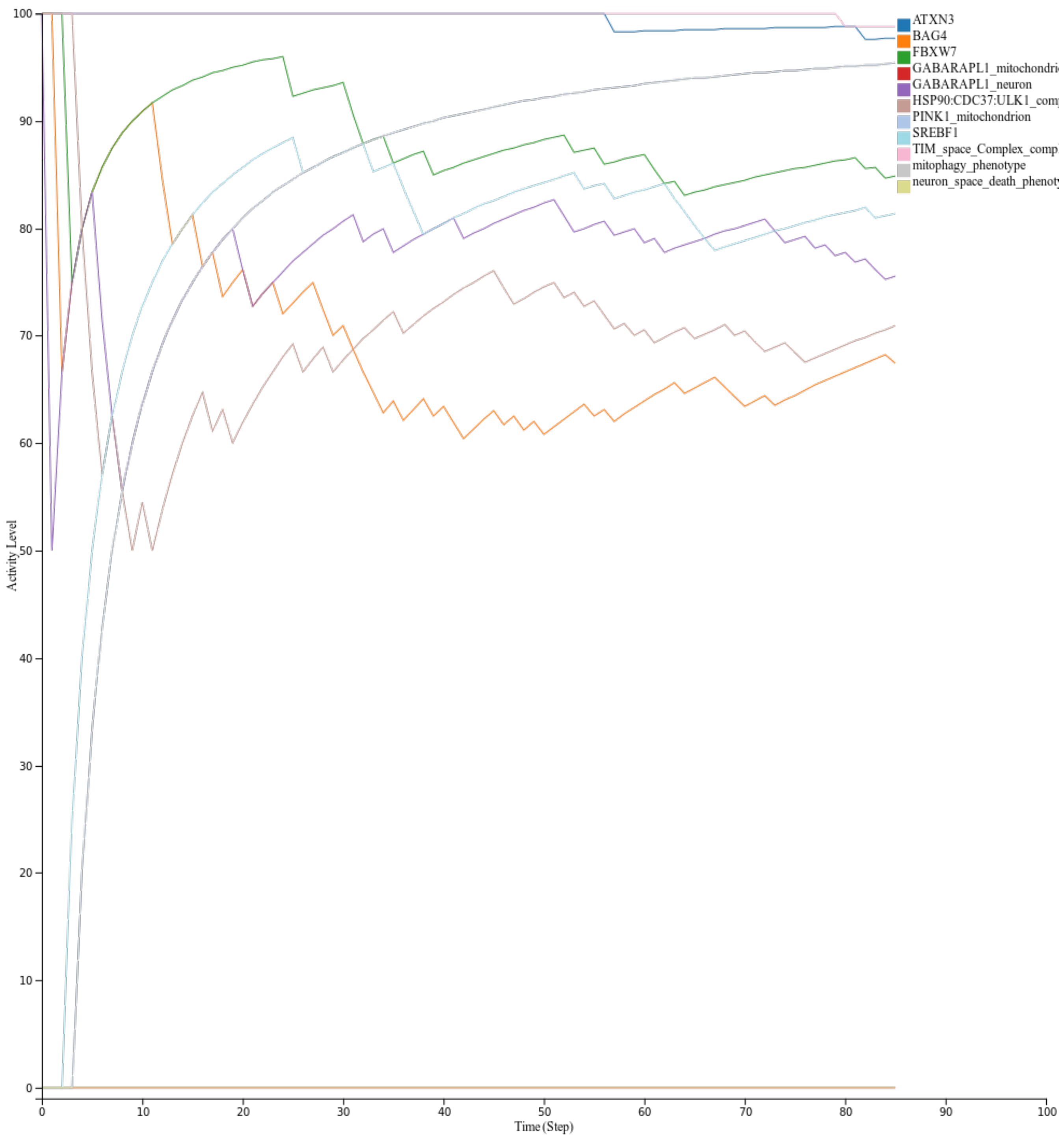

Figure S11: PRKN simulation

Pathway	Group ID	Identity-based distance	Similarity-based distance
Pi3k/akt	RPS6KB1	0.582524272	0.012230182
	PHLPP1	0.582524272	0.007517202
	WNT1	0.563106796	0.013361297
	WNT3	0.553398058	0.008483363
	PRKN	0.553398058	0.005372797
TCA cycle	PDP2:PDPR_complex	0.630769231	0.010729783
	alpha-ketoglutaratedehydrogenase_complex	0.630769231	0.009704142
	PDP1:PDPR_complex	0.6	0.01025641
	SIRT3	0.6	0.037790927
	isocitratedehydrogenase_complex	0.6	0.012544379
	GLUD1	0.6	0.01530572
PRKN	SREBF1	0.648148148	0.012003
	FBXW7	0.648148148	0.024005
	OPTN	0.648148148	0.012003
	HSP90	0.611111111	0.012003
PPARGC1A	IDH3G_rna	0.582089552	0.008539393
	TF_YY1_complex	0.582089552	0.060741071
	IDH3A_rna	0.582089552	0.008539393
	NDUFS8_rna	0.582089552	0.008539393
	ATP5MC1_rna	0.582089552	0.008539393
mTOR	TSC1:TSC2_complex_neuron	0.603174603	0.0095742
	SESN2	0.603174603	0.0095742
	NAMPT	0.587301587	0.009322247
	ROS	0.571428571	0.009070295
	Akt	0.571428571	0.009070295
Foxo3	MAP3K5	0.61971831	0.008728427
	EIF4EBP1_rna	0.605633803	0.008530054
	ATG12_rna	0.605633803	0.008530054
	BECN1_rna	0.605633803	0.008530054
	BBC3_rna	0.591549296	0.00833168
	JUN	0.577464789	0.008133307
	SIRT1	0.577464789	0.008133307
Dopamine transcription	EN1	0.852941176	0.013327206
	LRRK2	0.573529412	0.046243107
	FOXA2	0.573529412	0.008961397
	SNCA	0.558823529	0.022575827
	SFPQ	0.529411765	0.008272059
	PIN1	0.514705882	0.008042279
	RXRA	0.5	0.0078125

Table S1: Examples shows the significant distances between the original and perturbed attractors (Knockouts)

Pathway	Group ID	Identity-based distance	Similarity-based distance
Pi3k/akt	CTNNB1_phosphorylated	1	0.015081535
	EIF4EBP1_phosphorylated	1	0.017862192
	IRS1_phosphorylated	1	0.009708738
	CTNNB1_ubiquitinated_phosphorylated	1	0.009708738
	PDPK1	1	0.014209633
	RPS6KB1_phosphorylated	1	0.010085776
	AKT1_phosphorylated	0.990291262	0.018828353
	TFEB_complex	0.980582524	0.009685173
TCA cycle	2-oxoglutaricacid	1	0.035108481
	hydroxyglutaric_acid	1	0.033609467
	succinic_semialdehyde	1	0.025325444
	acetyl-CoA	1	0.016094675
	oxaloacetate_2	1	0.015384615
	succinyl-CoA	1	0.015384615
	succinic_acid	1	0.015384615
	GMP	0.984615385	0.014674556
	GTP	0.984615385	0.014674556
PRKN	PRKN_ubiquitinated	0.981481481	0.021604938
	PINK1_neuron	0.944444444	0.043552812
	ubiquitin_phosphorylated	0.944444444	0.017489712
	PGAM5__S_	0.907407407	0.016803841
	PINK1_mitochondrion	0.888888889	0.017489712
	PINK1	0.87037037	0.01611797
PPARGC1A	COX5A_rna	0.985074627	0.014702606
	COX7A2_rna	0.985074627	0.014702606
	COX5B_rna	0.985074627	0.014702606
	CYCS_rna	0.985074627	0.015296651
	complex_IV_complex	0.985074627	0.014702606
	SDHB_rna	0.985074627	0.014702606
mTOR	CAMKK2	0.634920635	0.010078105
	MAPK1_phosphorylated_phosphorylated	0.603174603	0.0095742
	AMPK_complex_neuron	0.571428571	0.009070295
	DDB1:CUL4A_complex	0.571428571	0.009070295
	PRKN_neuron	0.555555556	0.008818342
FOXO3	FASLG_rna	0.661971831	0.009323547
	MAPK9_phosphorylated	0.647887324	0.009125174
	FOXO3_neuron	0.591549296	0.00833168
	PPARGC1A_rna	0.591549296	0.00833168
	FOXO3_acetylated_phosphorylated	0.577464789	0.008133307
	BCL2L11_rna	0.577464789	0.008133307
	FIS1_rna	0.577464789	0.008133307
Dopamine transcription	ALDH1A1_rna	1	0.03125
	DRD2_rna	1	0.015625
	TH_rna	1	0.015625
	BDNF_rna	0.985294118	0.015395221
	DDC_rna	0.970588235	0.014820772
	SLC18A2_rna	0.941176471	0.014705882
	SLC6A3_rna	0.941176471	0.014705882
	PITX3	0.911764706	0.026711857
	TF_NR4A2_complex	0.897058824	0.242704504

Table S2: Examples shows the significant distances between the original and perturbed attractors (overexpressions)

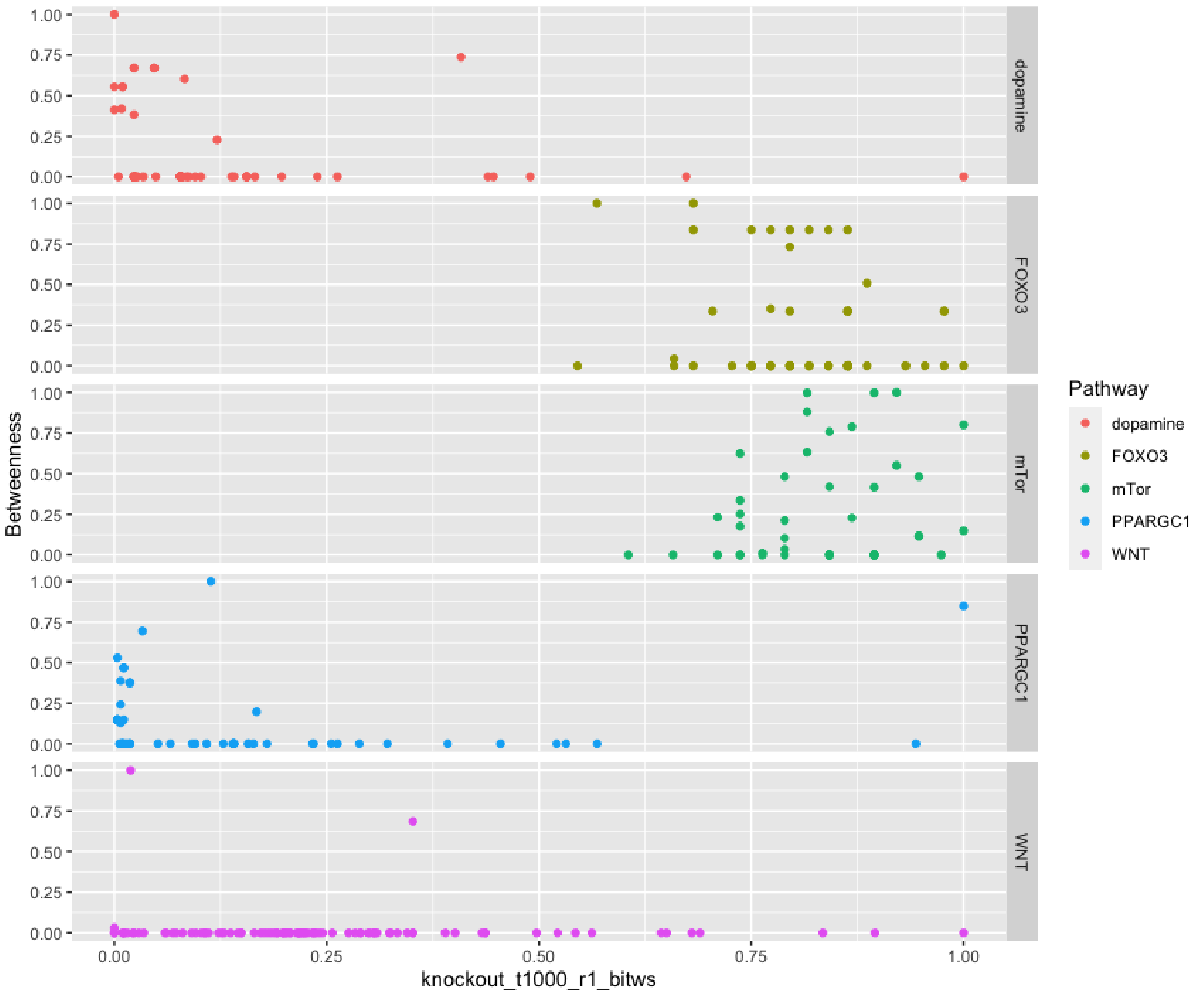

Figure S12: The figure represents molecules with high betweenness centrality and low knockout sensitivity in multiple pathways.

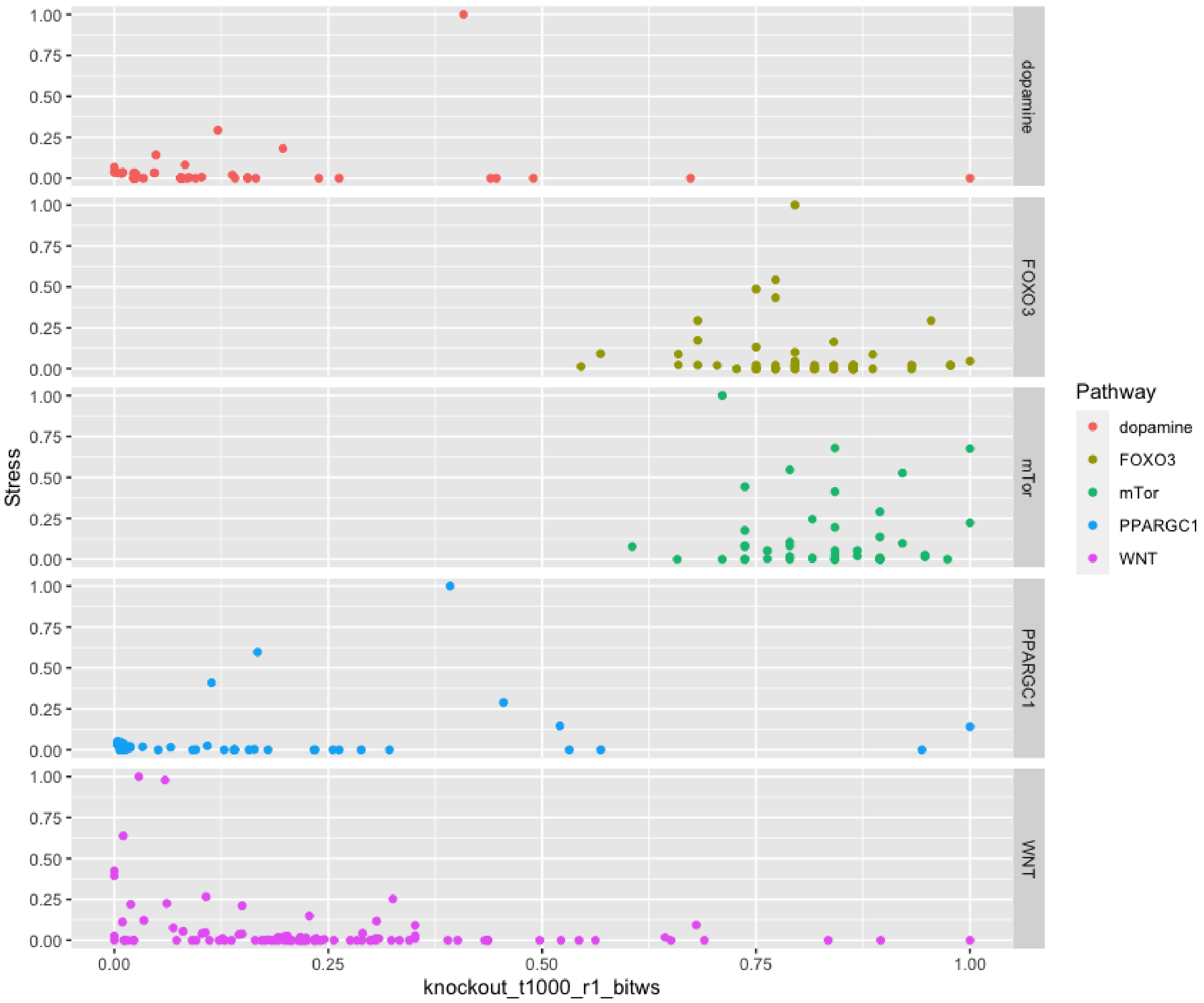

Figure S13: The figure represents the molecules with high stress centrality and low knockout sensitivities in multiple pathways suggests the presence of compensatory paths.

Pathway	Node ID	Degree			Betweenness	Stress
		Total	In	Out		
Dopamine transcription	TF NR4A2 complex	29	8	21	330	463
	PITX3	3	2	1	102	136
	TF PITX3 complex	13	2	11	66	84
	MIR133B rna	2	1	1	54	66
	RXRA	2	1	1	27	38
FOXO3 activity	TFFOXO3 complex nucleus	36	9	27	3673	9084
	TFCHOP:FOXO complex	4	2	2	1763	4932
	FOXO3 nucleus	3	2	1	1600	4416
	SIRT3	6	3	3	1482	3946
	BBC3 rna	3	2	1	896	2670
mTOR	AMPK complex neuron	10	7	3	2277	11064
	TSC1:TSC2 complex neuron	8	5	3	1465	7476
	STK11	6	5	1	1018	6050
	SESN2	5	2	3	788	2464
	nicotinamide	7	4	3	745	7518
PPARGC1A	PPARGC1A phosphorylated	13	4	9	322	650
	TF NRF1 complex	24	2	22	174	388
	PPARGC1A(AC-Ph)	4	3	1	94	188
	TF NRF2 complex	16	2	14	88	266
	TF YY1 complex	11	3	8	75	92
TCA cycle	2-oxoglutaricacid	25	19	6	451	761
	S-malate	12	7	5	195	490
	NADH	19	15	4	188	539
	ADP	15	8	7	178	393
	acetyl-CoA	10	6	4	152	384
	mTORC1 complex neuron	11	7	4	463	511
	AKT1 phosphorylated	7	5	2	440	500
Wnt-PI3K/AKT	PI3K	9	8	1	297	369
	PDPK1	2	1	1	260	326
	RPS6KB1 phosphorylated	5	3	2	189	201

Table S3: Common top five topological metrics in BMs and their source diagrams. The table summarises topological properties of nodes in the selected pathways, and “Node ID” indicate the specific nodes, “Degree” indicates a specific type of a node degree of in the BMs (total/incoming/outgoing connections), “Betweenness” describes node betweenness, while “Stress” describes the number of shortest paths that pass through a node

5 ATTRACTOR IDENTIFICATION

- The first step in identifying attractors is to define the Boolean model, which typically involves constructing a network of interacting nodes representing biological molecules or processes.

- Once the network is constructed, the model can be simulated using Boolean logic to determine the possible states or configurations of the system. The state of the system at each time step is determined based on the logical rules governing the interactions between the nodes in the network.

- The simulation is run over multiple time steps to explore the dynamics of the system and identify attractors, which are defined as stable states that the system can reach and maintain over time.

- One way to identify attractors is to use brute-force methods such as the SAT solver algorithm, which systematically explores all possible state combinations of the system to determine which ones are stable.

- Another approach is to use heuristic algorithms such as the heuristic method or the Monte Carlo method to simulate the system over time and identify attractors based on the frequency of states observed.

- Once the attractors are identified, they can be analyzed and compared to known biological states to validate their biological relevance.

- Additionally, the stability of the attractors can be assessed by perturbing the system and observing how the attractors change in response to these perturbations.

- Finally, the results can be interpreted and used to gain insights into the behavior of the biological system under study and to inform future experimental investigations.

The performance of asynchronous and synchronous simulations was evaluated in selected models to gain a comprehensive understanding of their characteristics and to determine their performance. The attractor analysis results indicated that the state trajectories converge to either fixed or cyclic attractors, dependent on the synchronous and asynchronous updating schemes. The comparison of four algorithms (HyTarjan, Heuristic, Decomp, and SAT) in terms of their calculation speed in pathways is summarized in table S4. A comparison of four different algorithms, namely HyTarjan, Heuristic, Decomp, and SAT, in terms of their calculation speed in pathways is presented in table S4.

The speed of the algorithms was compared. The SAT algorithm demonstrated improved time to find the attractors compared to the Decomp algorithm, with the exception of the TCA cycle. The SAT algorithm achieved a substantial 87.32% improvement in the ER stress signaling pathway, significantly reducing the time required to reach the attractor.

			Time (seconds)			
			Asynchronous		Synchronous	
Pathway	Edges	Targets	HyTarjan	Heuristic	Decomp	SAT
PGC1 alpha	109	PPARGC1A	3547	1789	173	96
		SIRT1	2587	1471	169	74
Dopamine transcription	167	NR4A2	2981	1460	147	54
Wnt/PI3K-AKT	391	Wnt/PI3K	1135	3961	403	256
ER stress signaling	53	DDIT3	971	1855	67	11
		AKDHC	2066	1123	84	110
TCA cycle	137	Oxoglutarate	2122	1140	84	110
		IDH	2153	1151	84	110
		SIRT3	2130	1140	84	110

Table S4: Attractor reachability speed in different algorithms. The table shows the duration of attracor reachabiliy for asynchronous and synchronous systems in the selected pathways, using the methods HyTarjan, Heuristic, Decomp, and SAT. The scales includes the node numbers, and Targets indicate the perturbed molecules

The landscape of the network state transitions along with attractor cycles were identified. The returned transition network object has same structures with the normal network object. The transition network is written as a SIF file. The SIF file could be loaded to Cytoscape with the following the steps in fig. S14.

The result is shown in fig. S15.

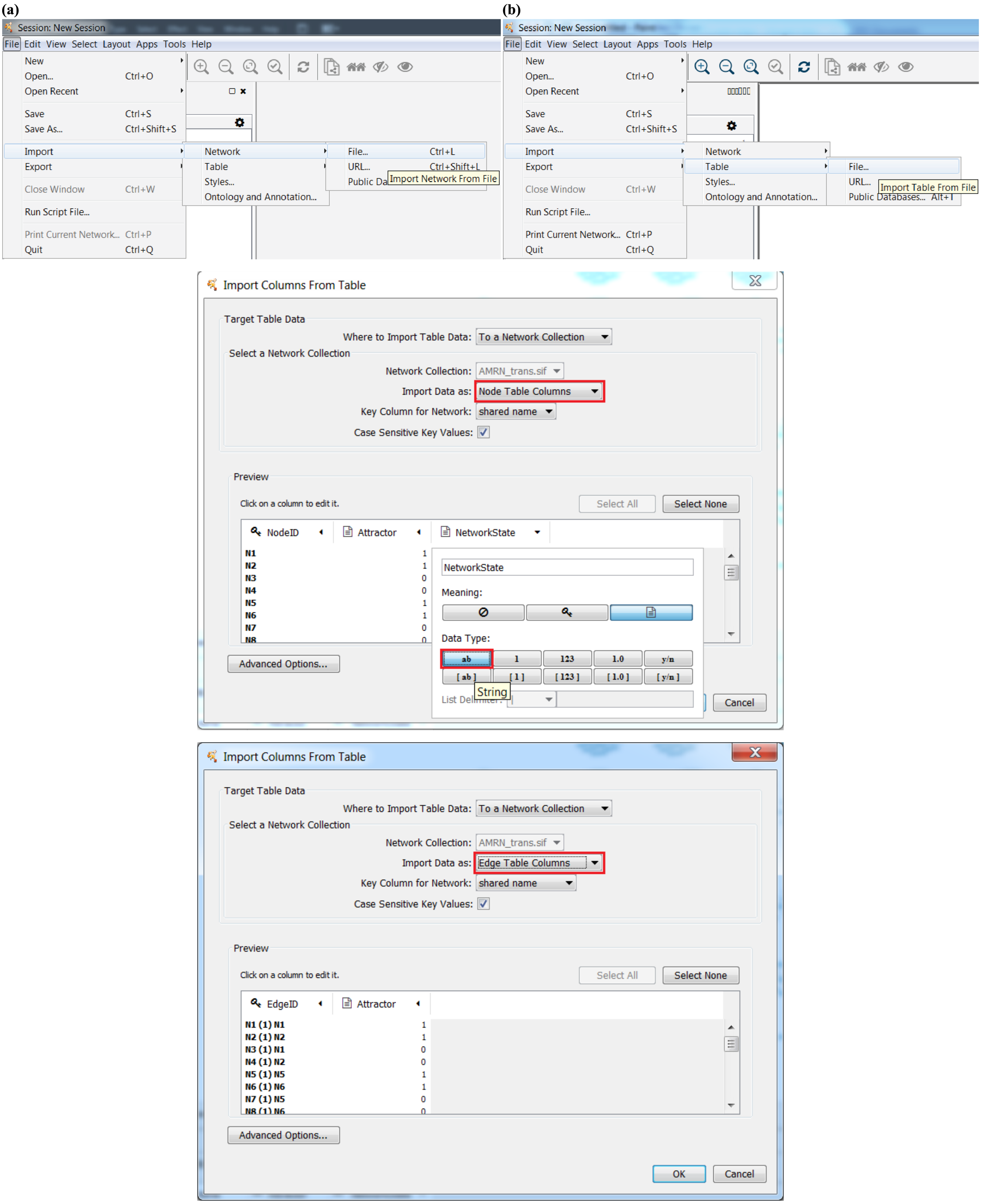

Figure S14: Steps for loading a SIF fyle in Cytoscape (top to bottom: opening the import dialogs, importing the nodes, importing the edges).

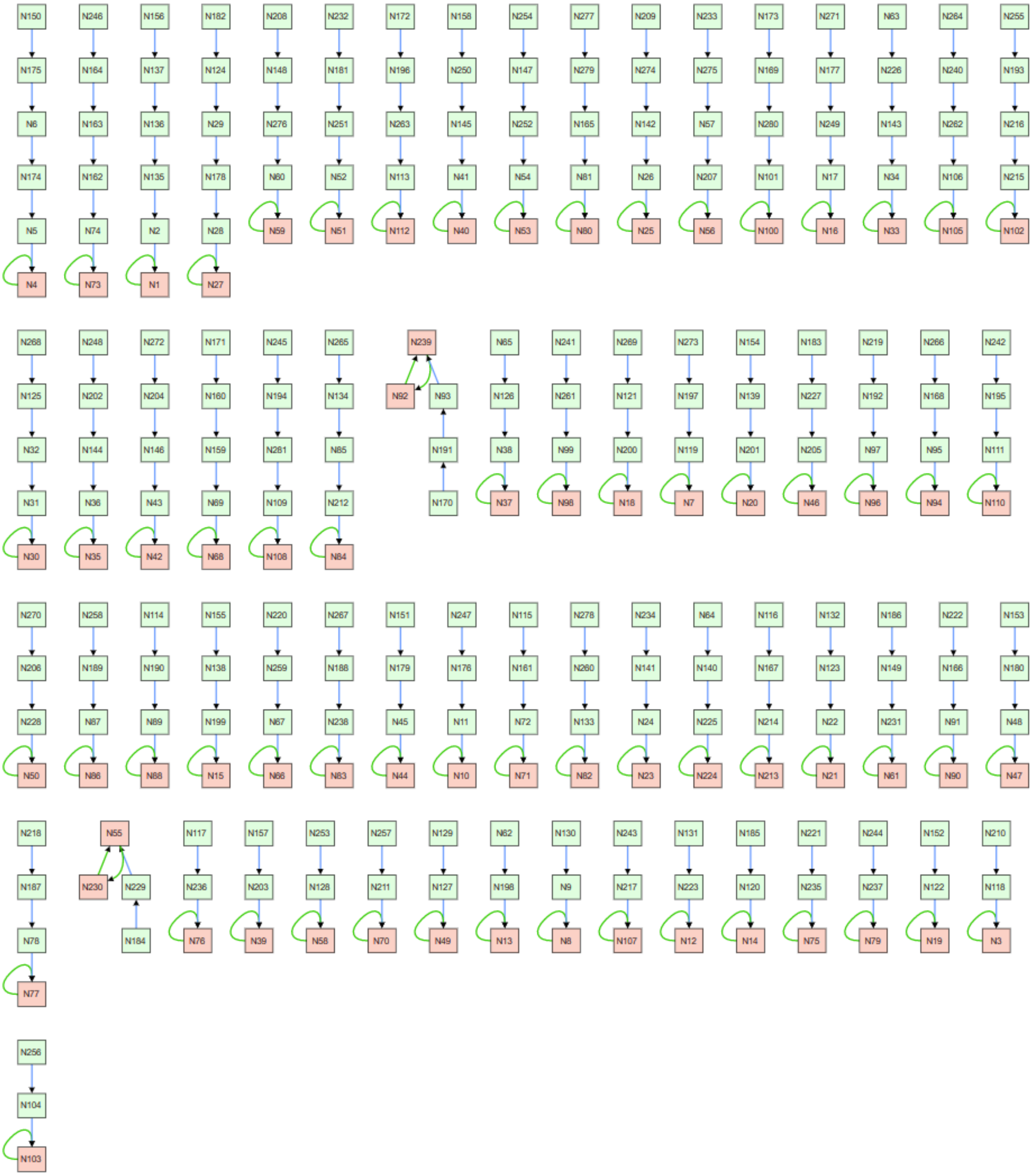

Figure S15: PPARGC1A pathway attractors: the figure represents a visualization of the identified attractors in the Boolean network. The attractors are represented as nodes and the edges between them represent the transitions between attractors. The attractors are colored according to their stability, with red indicating a stable attractor and green indicating an unstable attractor. The attractor values for each node are available in the stable GitLab repositories
